# Therapeutical Notes

**Published:** 1899-12-16

**Authors:** 


### THERAPEUTICAL NOTES.

### Local Applications in Rheumatism.

Arendt recommends ichthyol dressings for the affected joints, such as:—

℞. Ichthyol, ʒiiss.

Extracti belladonnæ, grs. xv.

Adipis Lanæ, ad ℥j.

M. fiat linimentum.

Or ℞. Ichthyol, ʒijss.

Alcohol, ʒijss.

Adipis Lanæ, ad ℥j.

M. ft. linimentum.

Another ointment found useful by Bourget, and praised by various authors, is:—

℞. Acidi salicyl., grs. xlv.

Olei terebenthini, ♏ xlv.

Adipis Lanæ.

Unguenta simplicis, āā ʒv.

M.

It is often noted that menthol or menthol and extract of belladonna can be substituted for the turpentine with advantage.—*Practitioner*, September.

The salicylic acid may also be applied as a collodion varnish, though it is not clear whether it is then absorbed. From the "Klin.-therap. Wochenschrift" we take another remedy:—

℞. Phenacetin, partes v.
Lanolini, partes xx.
Ol. olivarum, q.s.
M. ft. unguentum.

Or the drug may be dissolved in alcohol and applied under a hot compress.—*Medical Record*, July 8th.

### Broncho-Pneumonia of Children.

J. A. Coutts finds that severe cases such as follow measles are best treated by large doses of belladonna. The dyspnœa, pyrexia, and above all the mortality, are largely reduced, and the length of attacks is shortened. In several dozen cases at the Shadwell Hospital he could only recall two deaths. It reduces rapidly the secretion into the lungs, and is often given for that purpose in full doses before the administration of ether and other anæsthetics. It frequently prevents a fatal result in diphtheritic paralysis of the diaphragm by thus preventing effusion into the lungs.

For young children he gives the equivalent of 15 minims of the tincture (1898) every three or four hours, though he confesses that this caused slight delirium in two cases out of 60, and often some redness of the skin. Still, the striking benefit in broncho-pneumonia is so great that these disadvantages may be disregarded if the patient is carefully watched. — *Merck's Archives*, February 7th, 1899.

### Cold in the Head.

℞. Menthol,

Camphor, āā grs. v.

Alboleni, ℥ii.

M. S. Use with atomiser every two hours.

Max Nassauer asserts that an incipient cold can be stopped with a weak solution of potassium permanganate. The nose should be first blown, and then rinsed or syringed out with the solution, which should run out of the other nostril and into the throat. Afterwards a dry plug of cotton is pushed well up the nose, and the weak solution poured in, the head being held well back. The plug thus soaked is left for about an hour in position. At the beginning of an attack the effect is magical, but even later on good results are obtained.—*New York Medical Journal*, September 16th.

### Summer Diarrhœa.

Mons. Bizine ("Indépendance Médicale") recommends the tincture of iodine in an emulsion for summer diarrhœa. The mixture should be kept on ice to prevent decomposition if the weather is hot.

℞. Essent. menthæ piperis, ♏ v.

Essent. caryophylli, ♏ vii.

Tincture iodi, ♏ xv.

Chloroformi, ♏ v.

Emulsionis ol. Ricini, ℥vi.

Misce. S., ʒss. omne horâ.

If any looseness after two days still remains, he gives every four hours—

℞. Iodised starch, gr. ix.
Ft. pulvis.
—*New York Medical Journal*, May 16.

### Schleich's Mixtures for Anæsthesia.

The formula for these much-discussed mixtures is as follows:—

No. 1. Chloroform, 3 parts.
Petroleum ether, 1 part.
Sulphuric ether, 12 parts.

No. 2. Chloroform, 3 parts.
Petroleum ether, 1 part.
Sulphuric ether, 10 parts.

No. 3. Chloroform, 6 parts.

Petroleum ether, 1 part.

Sulphuric ether, 16 parts.

As there is a difficulty in obtaining a petroleum ether boiling at 60 deg. C., the officinal benzine is redistilled, and the lightest portions only are used. No 1 is used for short operations only, and the others for longer ones. About an ounce is necessary for a twenty minutes' anæsthesia.—*Merck's Archives*, June.

